# Monitoring of the Immune Dysfunction in Cancer Patients

**DOI:** 10.3390/vaccines4030029

**Published:** 2016-09-02

**Authors:** Saskia J. A. M. Santegoets, Marij J. P. Welters, Sjoerd H. van der Burg

**Affiliations:** Department of Medical Oncology, Leiden University Medical Center, 2333 ZA Leiden, The Netherlands; s.j.a.m.santegoets@lumc.nl (S.J.A.M.S.); M.J.P.Schoenmaekers-Welters@lumc.nl (M.J.P.W.)

**Keywords:** anti-tumor response, immune dysfunction, immunomonitoring, immunosuppression, phenotyping, functional assays, regulatory T cells, myeloid-derived suppressor cells, tumor-associated macrophages, tumor-associated neutrophils

## Abstract

Immunotherapy shows promising clinical results in patients with different types of cancer, but its full potential is not reached due to immune dysfunction as a result of several suppressive mechanisms that play a role in cancer development and progression. Monitoring of immune dysfunction is a prerequisite for the development of strategies aiming to alleviate cancer-induced immune suppression. At this point, the level at which immune dysfunction occurs has to be established, the underlying mechanism(s) need to be known, as well as the techniques to assess this. While it is relatively easy to measure general signs of immune suppression, it turns out that accurate monitoring of the frequency and function of immune-suppressive cells is still difficult. A lack of truly specific markers, the phenotypic complexity among suppressive cells of the same lineage, but potentially with different functions and functional assays that may not cover every mechanistic aspect of immune suppression are among the reasons complicating proper assessments. Technical innovations in flow and mass cytometry will allow for more complete sets of markers to precisely determine phenotype and associated function. There is, however, a clear need for functional assays that recapitulate more of the mechanisms employed to suppress the immune system.

## 1. Introduction

The common treatment modalities for curing cancer or decreasing tumor load are surgery, chemotherapy and radiotherapy. Recent clinical success has also been reported by a fourth therapy option, called immunotherapy. In cancer immunotherapy, the immune system is reinforced to fight cancer either by: (i) increasing the number of cytotoxic lymphocytes (for example, by vaccination, by adoptive transfer of autologous tumor-specific T cells, receptor engineered T cells or ex vivo-activated NK cells); (ii) activating the endogenous tumor-specific T cells by agonistic antibodies against co-stimulatory molecules (e.g., CD28, CD137, CD154, tumor necrosis factor receptor superfamily member 4 (OX40) and inducible T cell costimulator (ICOS)); (iii) blocking the checkpoint pathways that act on the anti-tumor T cells through administration of antibodies against co-inhibitory molecules (e.g., cytotoxic T lymphocyte antigen 4 (CTLA-4), programmed cell death protein 1 (PD-1), T cell immunoglobulin and mucin protein 3 (TIM3), V-domain Immunoglobulin suppressor of T cell activation (VISTA)); (iv) alleviation of the suppression induced by regulatory T cells (Tregs), myeloid-derived suppressor cells (MDSCs), tumor-promoting macrophages (so-called type 2 macrophages (M2) or TAMs) or type 2 tumor-associated neutrophils (N2 or TANs); and/or (v) by making use of monoclonal antibodies (mAbs) directly targeting tumor cells (e.g., CD20, HER-2, CD52 and epidermal growth factor receptor (EGFR)). Notably, these tumor-directed mAbs will not be discussed in this review. While many clinical successes are reported with these treatments, mitigation of immune cell suppression has been found to be tough.

Examples of clinically-successful immunotherapeutic approaches are especially found among treatments for melanoma. Major clinical improvement has been obtained by using checkpoint blocking antibodies against CTLA-4, PD-1 and PD-1 ligand (PDL-1), as well as their combination in advanced melanoma patients [1,2,3,4]. Similar success has been observed in other types of immunogenic cancers [5,6,7]. Furthermore, the adoptive cell transfer (ACT) of tumor antigen-specific T cells obtained and expanded from autologous tumor infiltrating lymphocytes [8,9] or peripheral blood mononuclear cells (PBMC) [10,11] has resulted in durable clinical responses and complete responses in late-stage melanoma patients. Another successful adoptive cell therapy is by transfer of genetically-engineered chimeric antigen receptors (CAR)-expressing cytotoxic T cells. CAR T cells express an extracellular tumor antigen recognition domain that is composed of a single chain variable fragment from an antibody and intracellular signaling and/or co-stimulatory domains allowing for T cell activation [12,13]. As an example, adoptive transfer of CD19-CAR T cells has resulted in remarkable clinical responses in patients with B cell malignancies [13,14]. Furthermore, reinfusion of ex vivo cytokine-activated haplo-identical natural killer (NK) cells has also led to promising anti-tumor responses in patients with hematological malignancies [15,16]. 

Beside passive infusion of relative high numbers of tumor-specific T cells, one can also actively stimulate the autologous T cell repertoire of patients, for instance through provision of agonistic antibodies directed to co-stimulatory molecules on T cells [17,18,19,20,21] or by injecting vaccines. Therapeutic cancer vaccines come in many flavors. Vaccine platforms include DNA, RNA, proteins or synthetic long peptides (SLP) encoding for tumor-specific or tumor-associated antigens, as well as vector-based vaccines (e.g., dendritic cell (DC) vaccines, bacterial and viral vectors, nanoparticles) [22,23,24]. Especially in the pre-malignant setting, we and others have shown that multiple vaccine platforms induced complete and partial regressions [25,26,27,28,29]. In the cancer setting, progress is slower. Examples of therapeutic cancer vaccines with clinical benefit include Sipuleucel-T immunotherapy for castration-resistant prostate cancer [30,31], the oncolytic herpes virus Talimogene Laherparepvec (T-VEC) for advanced melanoma patients [32,33], the granulocyte-macrophage colony-stimulating factor (GM-CSF) gene-transfected tumor cell vaccine (GVAX) used to prime combined with *Listeria monocytogenes*-expressing Mesothelin booster vaccine for metastatic pancreas cancer [34,35] and multiple tumor antigen-loaded autologous DC vaccines for advanced melanoma, hepatocellular carcinoma, malignant glioma and renal cell carcinoma patients [36,37,38]. 

**Figure 1:**
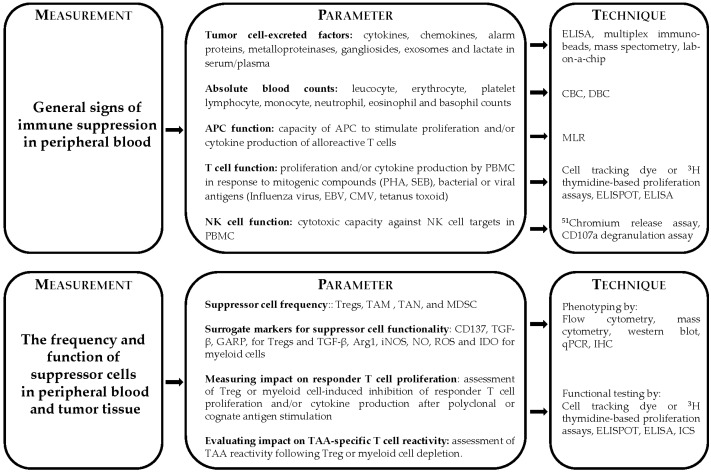
Overview and measurement of the different levels of immune dysfunction present in cancer patients. Immune dysfunction can be analyzed through measuring general signs of immune suppression in the blood (top left) and through measuring the frequency and function of suppressor cells in the blood and tumor tissue (bottom left). The different parameters that can be measured studying these levels of immune dysfunction are depicted (middle), as well as the techniques that can be used (right). Abbreviations used: APC: antigen-presenting cell; Arg1: arginase 1; CBC: complete blood count; CMV: cytomegalovirus; DBC: differential blood count; EBV: Epstein-Barr virus; GARP: glycoprotein A repetitions predominant; ICS: intracellular cytokine staining; IDO: indoleamine-2,3-deoxigenase; IHC: immunohistochemistry; iNOS: inducible nitric oxide synthase; MDSC: myeloid-derived suppressor cells; MLR: mixed lymphocyte reaction; NK: natural killer; NO: nitric oxide; PBMC: peripheral blood mononuclear cells; PHA: phytohemagglutinin; qPCR: quantitative polymerase chain reaction; ROS: reactive oxygen species; SEB: staphylococcal enterotoxin B; TAA: tumor-associated antigen; TAM: tumor-associated macrophages; TAN: tumor-associated neutrophils; TGF-β: transforming growth factor receptor-β; Treg: regulatory T cells.

These studies demonstrate that tumor-specific T cells can successfully combat cancer. However, tumors may escape from the immune system by strategies aimed at avoiding T cell recognition, including HLA downregulation [39] and/or the deletion, modification or decreased expression of tumor antigens [40]. Moreover, in many cases, cancer-associated immune suppressive networks may kick in to impair the induction, activation, expansion or the execution of the effector function of tumor antigen-specific T cells. Here, we briefly review the current knowledge on the different levels of this immune dysfunction present in cancer patients and discuss how to monitor this (Figure 1), as this will be important when new immunotherapeutic strategies are designed in which the alleviation of immune suppression is one of the goals.

## 2. General Signs of Immune Suppression

Tumor cells are capable of releasing various cytokines (reviewed in [41,42,43]), including interleukin-1 (IL-1), IL-6, IL-10, prostaglandin E2 (PGE2), transforming growth factor beta (TGF-β), vascular endothelial growth factor (VEGF), macrophage colony-stimulating factor (M-CSF), macrophage migration inhibitory factor (MIF) and granulocyte-macrophage colony stimulating factor (GM-CSF) [44,45,46,47]. In addition, tumor cells can also shed alarm proteins, such as the major histocompatibility complex (MHC) class I polypeptide-related sequence A (MICA) or B7-H6, thereby dampening NKG2D-mediated activation of T cells and NK cells or NKp30-mediated activation of NK cells [48,49]. Furthermore, tumor cells can release soluble factors, like chemokines, matrix metalloproteinases (MMPs) [50] and gangliosides [51], exosomes (small endosomal membrane vesicles that are enriched for tumor biomarkers, such as DNA, RNA and proteins [52]), as well as lactate [53], via which they can suppress immune responses. Interestingly, all of these tumor-derived factors can operate locally within the tumor microenvironment (TME), but also spread systemically via the blood compartment to mediate distant effects (e.g., on bone marrow, lymph nodes and spleen) and thus mediate systemic immune suppression. Many of these factors can be measured in blood plasma and/or serum and are, therefore, studied for their use as biomarkers for tumor progression or treatment efficacy, but they may also be used to obtain indications for immune suppression. 

Cytokine, chemokine and MMP levels are measured by ELISA, multiplex immunobead assays (i.e., Luminex or cytometric bead array) [54,55,56,57] or other assays, such as solid-phase proximity ligation assay or a combined immunoprecipitation and mass spectrometry assay [58,59]. Gangliosides can be detected in blood plasma by a chromatographic mass spectrometry-based assay [60], while antibodies against these gangliosides can be detected by ELISA. Tumor-derived exosomes can be isolated and characterized by novel state-of-the-art microfluidic lab-on-a-chip technology [61,62,63]. The secreted tumor-derived factors have been shown to impact lymphoid and myeloid subsets by skewing them towards immunosuppressive cells. They contribute to the inhibition of effector T cells and the induction of Tregs [64], as well as to the differentiation of myeloid cells into tolerogenic DCs [65,66,67], TAMs [68], MDSCs [69,70] or the attraction and/or induction of TANs [71,72] that share features with MDSCs [72]. Significant negative correlations between serum levels of IL-6 [73,74], TGF-β [75,76] and IL-10 [77] and disease-free and/or overall survival were observed in multiple cancer types, suggesting that the measurement of these soluble factors can be used to assess whether immune suppression can be expected.

Another parameter that can be used to assess general immune dysfunction in cancer patients is the abnormal frequencies of certain types of immune cells in blood. Abnormal high counts of leukocytes (leukocytosis) are one of these. Leukocytosis is observed in patients with colorectal cancer [78], lung cancer [79], cervical cancer [80] and ovarian cancer [81] and served as an indicator of poor prognosis in these patients. In the blood of advanced-stage cervical cancer patients, the number of monocytes were found to be much higher compared to those in healthy donors, and this was associated with immune suppression [82]. Similar observations were made in patients suffering from different types of cancer [83,84,85,86]. Alternatively, leukopenia, which is decreased numbers of leukocytes (or subsets), has also been observed in some types of cancer (e.g., head and neck, colon, lung cancer and Hodgkin lymphoma) [87,88]. Differential cell counts in whole blood, which are routinely done in every hospital, can reveal whether the total leukocyte, as well as the subset cell counts are within the normal range or are altered. Moreover, the lymphocyte to monocyte ratio, the platelet to lymphocyte ratio or others have been shown to be associated with the response to therapy and/or clinical outcome [89]. Most recently, it was found that an increased absolute number of lymphocytes [90] or a low neutrophil to lymphocyte ratio in the blood [91] were associated with less suppression and a better response to checkpoint therapy. 

There are also functional approaches to assess systemic immune suppression through the analysis of general antigen-presenting cell (APC), T cell and NK cell function. The capacity of circulating APCs to stimulate T cells can be easily tested using a mixed lymphocyte reaction (MLR). In this assay, the capacity of APC to stimulate proliferation and cytokine production of alloreactive HLA-mismatched T cells is assessed [82,92]. In pancreatic cancer patients, the circulating DCs were not only found to be decreased in number, but also their stimulatory function was impaired when measured by MLR [93]. Furthermore, in other types of cancer, the functional capacity of DCs was found to be impaired [94,95,96,97]. To test the functional capability of circulating T cells, PBMC can also be tested for their responsiveness to activating signals. General T cell function can be assessed by analyzing the proliferation and/or cytokine production of PBMC by mitogenic (chemical) compounds like phytohemagglutinin (PHA) [98] and staphylococcal enterotoxin B (SEB) [99] in a non-HLA restricted and APC-independent manner. A more subtle approach to test T cell functionality is by analyzing T cell proliferation and/or cytokine production in response to common bacterial and viral antigens, the so-called recall antigens. Examples of such recall antigens are proteins derived from influenza virus, cytomegalovirus (CMV), Epstein-Barr virus (EBV), tetanus toxoid, *Mycobacterium tuberculosis* and *Candida albicans*. Alleviation of suppression, as measured by improved T cell function (either by increased proliferation or cytokine production) against recall antigens, could be observed in cancer patients upon anti-tumor therapy [82,100,101]. Of note, these analyses can give valuable information on the level of T cell suppression, as the absence of T cell responsiveness following the strong mitogenic PHA stimulation may reveal T cell intrinsic problems, and the absence of recall antigen-specific responses may be indicative of a state of more general tumor-induced immune suppression. To test the functional activity of circulating NK cells, which is often reduced in patients with cancer [102,103], PBMC can be tested for their cytotoxic activity against NK cell targets (i.e., MHC-devoid targets, such as K562 cells) by the standard ^51^chromium release assay or CD107a (lysosome-associated membrane protein 1 (LAMP-1)) flow cytometric degranulation assay [104]. 

## 3. Immune Dysfunction through the Induction of Suppressor Cells

The role of lymphoid and myeloid suppressor cells in tumor development and progression has been studied extensively over the past decades [64,68,69,105,106]. By making use of cell-depleting agents or conditional cell ablation models based on the diphtheria toxin receptor, the role and contribution of specific immune cell subsets in the suppression of anti-tumor immune responses have been revealed in preclinical settings. Ablation of Tregs can result in dramatic tumor reduction and/or complete tumor clearance of large established tumors [107,108,109]. Similarly, the suppressive role of MDSC, TAM and TAN have also been demonstrated [110,111,112,113,114], emphasizing that several types of immune cells play an important role in suppressing an (initially) effective anti-tumor response. 

Obviously, it is much harder to study the role of lymphoid and myeloid suppressor cells in human beings. Generally, the functional impact of such cells is determined by the association in that the frequency of certain phenotypic populations of immune cells is increased in the blood or tumor of patients with a higher stage of disease or in patients with a worse immunological response or clinical outcome. A major obstacle in this type of analysis is that the unambiguous enumeration of these immunosuppressive cell subsets is hampered by the absence of exclusive, highly specific markers for functionally-active cells. While in mice, specific markers for MDSC and Treg detection have been identified (Gr-1 and its isoforms Ly6C and Ly6G for MDSC and Foxp3 for Treg detection), in humans, the identification of these cells is more complex, as Gr-1 is not expressed on human leukocytes [115], and Foxp3 can also be expressed on activated non-regulatory T cells [116,117]. As a result, a multitude of human MDSC and Treg subsets with different phenotypes has been documented in several types of tumors in the last decades [118,119]. As an example, a recent in-depth phenotypic analysis of human Tregs revealed 22 distinct subpopulations [120], while the myeloid cell subpopulations exceeded one hundred [121]. This makes correct interpretation of data and comparison between studies difficult. To tackle the heterogeneity in current human MDSC and Treg phenotyping panels, proficiency panels and workshops aiming at harmonization of their detection through developing robust marker combinations and gating strategies are being performed [122,123]. 

So far, there were a number of studies showing that significantly higher levels of Tregs [124,125,126,127], MDSC [90,128,129,130,131], (tumor-associated) macrophages [85,132,133] and neutrophils [105,134,135] could be detected in the peripheral blood and TME of almost all types of cancer, merely in advanced stages of the disease, and these high levels usually negatively correlated with clinical outcome and/or survival. 

Despite advances in the formulation of essential marker sets and gating strategies for such analyses, data on their functionality is still lacking and, as such, the link between phenotype and function. Since functional assessment of immune suppressor cells in the TME usually is not feasible due to limited tissue material, more in-depth analysis of (surrogate) markers for immune suppressor cell functionality would be an attractive approach to gain some more insight into their suppressive capacity. Examples of such markers include arginase 1 (Arg1), inducible nitric oxide synthase (iNOS), reactive oxygen species (ROS), TGF-β, indoleamine 2,3-dioxygenase (IDO) and IL-10, all of which can be expressed by myeloid suppressor cells [70,105,136]. The T cell-suppressive factors Arg1, ROS and IL-10 can also be produced by tumor-promoting TANs [137]. In addition, they can produce macrophage inflammatory protein 1-alpha (MIP-1α or CCL3), which recruits more myeloid cells into the tumor bed [70,72,105]. 

The activity of the enzyme Arg1 leads to increased L-arginine (L-arg) metabolism and converting it into ornithine and urea [138,139]. This way, it results in the deprivation of L-arg for the T cells, which is required for their proliferation and metabolism [140], and subsequently leads to impairment of T cell function [141,142]. Another enzyme involved in the metabolism of L-arg is iNOS, which converts L-arg into nitric oxide (NO) and citrulline. NO can mediate direct effects, but it can also be converted into peroxynitrite when ROS is available [143,144]. Peroxynitrites, which are secreted by MDSCs, can alter the TCR complex through inducing posttranslational modifications, dissociation of the TCR or selective degradation of the ζ-chain and, thereby, inhibit downstream signaling and T cell effector function [70,145]. This effect requires antigen presentation by MDSC, as in double transgenic mice, in which one T cell can recognize two antigens, it has been shown that the reactivity of T cells was only lost to the epitopes presented by the MDSC and not against the other antigen [145]. Notably, this shows that the MDSC can also suppress in an antigen-specific manner. ROS can also desensitize the TCR by inducing loss of TCR ζ-chain expression [146]. IDO exerts its suppressive effect by depletion of local L-tryptophan, thereby inhibiting T cell activation and proliferation, as well as inducing Tregs [147]. Arg1, iNOS and IDO expression can be detected by flow cytometry [148,149,150,151,152], Western blot [153], immunohistochemistry (IHC) [153] or quantitative PCR (qPCR) [154,155,156]. Arginase activity can be measured in cell lysates through measuring urea production [157,158]. ROS production can be measured by flow cytometry using an oxidation-sensitive dye named dichlorodihydrofluorescein diacetate (DCFDA) [148,155]. TGF-β and IL-10 are mostly detected by qPCR [156,159,160].

In the majority of laboratories, flow cytometry is generally limited to a maximum of 18 parameters (setting aside some experimental machines), and therefore, incorporation of these functional markers into existing flow panels is often not feasible. Currently, the novel multi-parameter single cell analysis technology named mass cytometry, or cytometry time-of-flight (CyTOF), would be an attractive alternative platform for incorporation of functional markers. CyTOF technology combines mass spectrometry with flow cytometry methodology. It utilizes target antibodies labeled to rare heavy metals for the simultaneous detection of up to 40 parameters [161,162,163] and, thus, permits a more in-depth single cell analysis. CyTOF’s ability to allow for in-depth profiling makes it a very attractive approach when complex and extensive immunophenotyping is desired.

A major drawback of using flow cytometry or CyTOF analysis is that it is a single cell analysis and, thus, does not provide any information about their location and functional orientation within the TME. A technique that holds great promise for the analysis of TME is multiplex IHC. Multiplex IHC permits the identification of up to 30 antigens on a single tissue slide or a group of different tissue samples (reviewed in [164]). Examples of such techniques include the multiplexed IHC combined with multispectral imaging system (Vectra platform [165]), sequential multiplex staining making use of repetitive staining and erase techniques [166,167] or image mass cytometry using a combination of mass cytometry, IHC and immunocytochemistry techniques [168]. These approaches are especially attractive because they allow the analysis of the TME on archival, paraffin-embedded tissue samples, and they retain the complete tumor (immune) contexture, i.e., this permits analysis of the location, density and functional orientation of the immune cell populations within the tumor and its surrounding stroma.

Taken together, the current and emerging monitoring strategies are well suited to study lymphoid and myeloid suppressor cells, especially at the level of quantification and immunophenotyping. Yet, one has to take into account that most of the evidence on the clinical relevance of these cells in suppressing anti-tumor immunity in humans was based on association and correlation and not based on formal proof, as can be obtained in murine studies. Therefore, we feel that it is of utmost importance that the functional capability of these suppressor cells be also analyzed. 

## 4. Monitoring Functional Capacity of Lymphoid and Myeloid Suppressor Cells

### 4.1. Monitoring Suppressive Capacity of Tregs

Given their potential suppressive role in anti-tumor immunity, Tregs are often monitored in clinical (immunotherapy) trials. In the majority of studies, Treg assessment is restricted to phenotypic characterization and quantification. Yet, the absence of exclusive and highly specific Treg markers in humans and the presence of a high number of distinct subpopulations [120] complicates the analysis, leads to a multitude of reported phenotypic Treg definitions, as well as to a blurred picture regarding the association between Tregs and clinical outcome (reviewed by Whiteside et al. [117,119]). Especially since information on the suppressive capacity of the identified Treg population often is lacking in these studies, assessment of the suppressive capacity of each of the identified Treg populations, therefore, seems essential in the context of immunomonitoring.

The study of antigen-independent in vitro suppression of T cell reactivity was one of the first assays to functionally study the immune suppressive function of Tregs. It measures the inhibition of responder T cell proliferation, either through analyzing the dilution of cell tracking dyes (i.e., carboxyfluorescein succinimidyl ester (CSFE) and Cell Trace proliferation reagent) or by 3H-thymidine uptake by proliferating cells. In general, responder T cells are polyclonally stimulated (i.e., via soluble, bead- or plate-bound anti-CD3 ± anti-CD28 antibodies) and co-cultured with Tregs at different ratios [169,170,171,172]. Unfortunately, this approach requires large numbers of isolated Tregs, which are often not available in the context of clinical trials. A major advantage of measuring 3H-thymidine uptake over the dilution of tracking dyes is that it displays good sensitivity to detect suppression with low numbers of Treg cells [173]. In fact, a recent protocol by Tree and co-workers [172] permits Treg suppressive analysis with just 10,000 isolated Treg, illustrating that this assay could be used for immunomonitoring studies. Interestingly, this assay could be done in an APC-independent (with anti-CD3/anti-CD28-beads) and APC-dependent (autologous B cells + PHA) fashion. Studies in our lab confirmed the applicability of this assay for monitoring Treg suppressive function in cancer patients. Moreover, we were able to show that unlike the CFSE-based flow cytometric proliferation assay, this 3H-thymidine based assay is capable of detecting the suppressive function of healthy donor-derived Tregs, confirming that indeed the 3H-thymidine-based approach is much more sensitive. 

Later, also antigen-dependent Treg suppression assays were developed as in the above-mentioned in vitro suppression assays, as quite a robust activation of responder T cells was provided by agonistic antibodies to CD3 and CD28, while this does not really reflect the cognate interactions between APC and T cells. Therefore, in an alternative setting, the suppressive effect of Tregs can be analyzed through evaluating their impact on tumor-associated antigen (TAA)-specific T cell reactivity in vitro. Using this setup, Tregs were found to suppress multiple tumor-specific responses in assays where Tregs were depleted prior to the assessment of tumor antigen-specific T cell reactivity in patients with urothelial cancer or colorectal cancer [174,175,176]. Similarly, the addition of Tregs to T cell cultures in which the effector cells specifically were stimulated with cognate antigen was also found to block antigen-specific effector function [177].

When it was realized that tumor-infiltrating Tregs are able to recognize tumor-derived antigens and can be expanded by cancer vaccines [178,179,180], assays that allowed the assessment of the suppressive capacity of these antigen-specific Tregs were developed. To test the suppressive effect of antigen-specific Treg cells, antigen-activated regulatory T cells can be co-cultured with either polyclonally-activated or antigen-specifically-activated responder cells, after which the inhibition of T cell proliferation, activation and/or cytokine production can be analyzed through different flow cytometric approaches, including activation marker expression, CFSE dilution for proliferation and cytokine production (IFN-γ, IL-2). Indeed, inhibition of responder cell proliferation could be shown upon stimulation with the cognate antigen employing this methodology [174,177,181].

Current approaches focus on direct suppression of effector T cells by Tregs as if it were one type of functional cell. Recent findings indicate that there is a high phenotypic and functional diversity in human Treg cells [120]. Moreover, different Treg subsets can be in different states of maturation, differentiation and activation (reviewed in [182,183,184]) and have distinct homing capabilities [185], suppressive mechanisms [186] and targets for suppression [187,188,189]. In addition, it has been shown that distinct subsets of Tregs can effectively regulate different types of Th cell responses in vivo [190]. This illustrates that it is very important to gain a full understanding of what Treg subset (phenotype) targets what type of immune responses, in which disease and by what mechanism, if one wants to perform the assay that is suitable for that situation. This requires the development of new and alternative methodologies. In the last couple of years, potential new ways to determine antigen specificity and activation status of Tregs have been developed. A candidate marker for the identification of such activated/antigen-specific Tregs is CD137 [191,192]. As described, combined CD137 and CD154 analysis following short-term (6 h) stimulation allows for the discrimination between CD137+CD154− Tregs (with stable Foxp3 expression) and CD137+CD154+ activated T cells (with instable Foxp3 expression) [191]. Alternatively, the transmembrane protein glycoprotein A repetitions predominant (GARP) has also been described to selectively identify activated human Foxp3+ Tregs following T cell receptor (TCR) stimulation [193,194]. Whether these markers will also be useful to identify antigen-specific Tregs ex vivo in human blood or tissue samples still needs to be elucidated. 

### 4.2. Functional Analysis of Myeloid Cell Suppression

Myeloid cells with suppressive capacity are a heterogeneous population of immune regulatory cells originating from the common progenitor myeloid lineage that is arrested in differentiation to DCs, macrophages or granulocytes [195,196,197,198]. Their normal function is to control immunopathology, as they expand in the bone marrow and/or spleen upon infections, stress, trauma and other pathological conditions, such as cancer [199,200,201]. Analysis of the suppressive function of myeloid cells is still in its starting phase, since it is not known exactly what, when and how the suppression by myeloid on effector cells should be measured due to the heterogeneity of this cell population. It is not yet clear whether their suppressive capacity in vivo depends on cell-cell interaction or acts via the production of soluble factors that interfere with the function of anti-tumor effector cells (i.e., T cells, NK cells and B cells [106]). 

Suppression factors produced by MDSC that have been identified are the aforementioned Arg1, iNOS, ROS and IDO, but also IL-10 and TGF-β [141,202]. Already almost 20 years ago, HLA-DRlow/-CD16+CD54+CD14+ IL-10-producing monocytes that were able to suppress autologous T cells were identified in ascites of ovarian cancer patients [203]. Moreover, TGF-β, and not Arg1 or iNOS, was shown to be involved in the suppressive capacity of CD14+HLA-DRlow/− cells obtained from melanoma patients [204]. 

Because of the lack of specific functional markers on myeloid-suppressive cells, it is more accepted to functionally access the capacity of these cells isolated from blood, bone marrow or TME in an in vitro cell-based assay. Whether close proximity or cell-to-cell contact is important for the suppressive efficacy of the myeloid cells is difficult to identify in in vitro assays, except for those earlier mentioned soluble mediators for which blocking antibodies exist. In a classic functional assay, isolated suppressive myeloid cells are co-cultured with activated responder T cells, and inhibition of the proliferation (often with 3H-thymidine incorporation or the flow-based technique) and cytokine production by these responder T cells is measured [143,205,206]. Using this assay, the responder T cells are often activated by anti-CD3/anti-CD28 beads or antibodies, which is not always mimicking the physiological situation in the tumor [207,208]. In melanoma patients, it was demonstrated that the proliferation and cytokine production (IFNγ) of autologous non-specific stimulated T cells were suppressed by 40% at the physiological ratios of CD14+HLA-DR-/low MDSC to T cells (1:4) [209]. Further analysis revealed that the suppressive molecules Arg1 and ROS were partly responsible for the suppressive effect. More importantly, cell-to-cell contact or close cell proximity was a prerequisite for this effect, as the suppressive capacity of the MDSCs was prevented in trans-well experiments [157,209]. 

l-arg deprivation and peroxynitrites can lead to loss or downregulation of CD3/ζ-chain (C247), which is part of the TCR complex in T cells and of the NKp46, NKp30 and CD16 killing receptor complexes on NK cells and, therefore, to the non-responsiveness of these two types of effector cells [210,211]. The expression of CD247 can be measured by quantitative PCR or flow cytometry. In advanced stage NSCLC patients, L-arginase- and iNOS-producing CD11b+CD14−CD15+CD33+ MDSCs were shown to downregulate CD247 on CD8+ T cells, thereby suppressing their function [212]. Downregulation of CD247 and high serum levels of nitric oxide (NO) and ROS are associated with high frequencies of circulating MDSCs and worse prognosis for cancer patients [106]. 

We have demonstrated that the depletion of CD14+ myeloid cells from the PBMC of patients with advanced cervical cancer or lung cancer resulted in the increase of T cell reactivity against recall antigens, but also unmasked tumor-specific T cell reactivity [82,213], albeit that the mechanism of action was not studied. 

Most of the functional assays conducted with myeloid-suppressive cells focus on the suppression of effector T cells; however, in vivo, particular subsets of myeloid cells might (preferentially) suppress other immune cells, like DCs [214], macrophages [215,216], NK cells [217], Tregs [218] and B cells [106]. Moreover, often, circulating and bone-marrow-derived myeloid suppressor cells are used for studying their functional capabilities [128], whilst the tumor infiltrated or resident myeloid cells may have different suppressive potential. Indeed, in melanoma patients, CD11b+lineage− MDSCs isolated from the blood were able to suppress T cell proliferation, while the tumor infiltrating myeloid cells were not [219]. In contrast, CD11b+CD14− granulocytic MDSCs obtained from head and neck cancer (HNC) in patients were able to suppress non-specific stimulated T cells, while their blood-derived counterparts were not [158]. In addition, Corzo and co-workers also showed that MDSCs from peripheral blood (HNC patients) or lymph nodes (tumor-bearing mice) only suppressed tumor-specific T cell responses, but not the responsiveness of T cells to non-specific stimulation, while MDSCs isolated from tumors suppressed both types of T cell responses [158]. Altogether, this shows that the MDSC can suppress in an antigen-specific manner and that the type of suppression may depend on the compartment from where the MDSCs are obtained (i.e., periphery, lymph node, TME). Whereas harmonization of phenotypical analysis of MDSCs is going on [122], even more effort should be undertaken to also obtain consensus about the functional assays. 

## 5. Conclusions 

The measurement of immune dysfunction is important to improve current immunotherapeutic approaches and for the design of new strategies to alleviate immune suppression. This still requires a huge effort with respect to setting-up reliable phenotypic and functional monitoring tools of the suppressive cells, which are correlated with the clinical outcome of the cancer patients. When successful, this might lead to a higher percentage of patients who benefit from the treatment, as well as increase the duration of the clinical response. 

## Abbreviations

The following abbreviations are used in this manuscript:

ACT	adoptive cell transfer
APC	antigen-presenting cell
Arg1	arginase 1
CAR	chimeric antigen receptor
CFSE	carboxyfluorescein succinimidyl ester
CMV	cytomegalovirus
CRC	colorectal cancer
CTC	circulating tumor cell
CTLA-4	cytotoxic T lymphocyte antigen 4
CyTOF	cytometry time-of-flight
DC	dendritic cell
EBV	Epstein-Barr virus
GM-CSF	granulocyte-macrophage colony stimulating factor
HPV	human papillomavirus
ICOS	inducible T cell co-stimulator
IDO	indoleamine-2,3-deoxigenase
IHC	immunohistochemistry
IL	interleukin
iNOS	inducible nitric oxide synthase
l-arg	l-arginine
M-CSF	macrophage colony stimulating factor
MDSC	myeloid-derived suppressor cells
MHC	major histocompatibility complex
MICA	MHC class I polypeptide-related sequence A
MIF	macrophage inhibitory factor
MLR	mixed lymphocyte reaction
MMP	matrix metalloproteins
NK	natural killer
NO	nitric oxide
OX40	tumor necrosis factor receptor superfamily member 4
qPCR	quantitative polymerase chain reaction
PBMC	peripheral blood mononuclear cells
PD-1	programmed cell death protein 1
PDL-1	PD-1 ligand
PGE2	prostaglandin E2
PHA	phytohemagglutinin
ROS	reactive oxygen species
SEB	staphylococcal enterotoxin B
SLP	synthetic long peptide
TGF-β	transforming growth factor receptor-β
Treg	regulatory T cells
TAM	tumor-associated macrophages
TAN	tumor-associated neutrophils
TCR	T cell receptor
TIM3	T cell immunoglobulin and mucin protein 3
TME	tumor micro-environment
T-VEC	Talimogene Laherparepvec
VEGF	vascular endothelial growth factor
VISTA	V-domain immunoglobulin suppressor of T cell activation
